# One Social Media Company to Rule Them All: Associations Between Use of Facebook-Owned Social Media Platforms, Sociodemographic Characteristics, and the Big Five Personality Traits

**DOI:** 10.3389/fpsyg.2020.00936

**Published:** 2020-05-29

**Authors:** Davide Marengo, Cornelia Sindermann, Jon D. Elhai, Christian Montag

**Affiliations:** ^1^Department of Psychology, University of Turin, Turin, Italy; ^2^Department of Molecular Psychology, Institute of Psychology and Education, Ulm University, Ulm, Germany; ^3^Department of Psychology, The University of Toledo, Toledo, OH, United States; ^4^Department of Psychiatry, The University of Toledo, Toledo, OH, United States

**Keywords:** Facebook, Instagram, WhatsApp, individual differences, Big Five personality traits

## Abstract

Currently, 2.7 billion people use at least one of the Facebook-owned social media platforms – Facebook, WhatsApp, and Instagram. Previous research investigating individual differences between users and non-users of these platforms has typically focused on one platform. However, individuals typically use a combination of Facebook-owned platforms. Therefore, we aim (1) to identify the relative prevalence of different patterns of social media use, and (2) to evaluate potential between-group differences in the distributions of age, gender, education, and Big Five personality traits. Data collection was performed using a cross-sectional design. Specifically, we administered a survey assessing participants’ demographic variables, current use of Facebook-owned platforms, and Big Five personality traits. In *N* = 3003 participants from the general population (60.67% females; mean age = 35.53 years, SD = 13.53), WhatsApp emerged as the most widely used application in the sample, and hence, has the strongest reach. A pattern consisting of a combined use of WhatsApp and Instagram appeared to be most prevalent among the youngest participants. Further, individuals using at least one social media platform were generally younger, more often female, and more extraverted than non-users. Small differences in Conscientiousness and Neuroticism also emerged across groups reporting different combinations of social media use. Interestingly, when examined as control variables, we found demographic characteristics partially accounted for differences in broad personality factors and facets across different patterns of social media use. Our findings are relevant to researchers carrying out their studies via social media platforms, as sample characteristics appear to be different depending on the platform used.

## Introduction

Currently, about 2.7 billion individuals use at least one of the Facebook-owned platforms (Newsfeed.org, 2019). With 2.4 billion current users, Facebook still represents the platform with the largest outreach, followed by WhatsApp (1.6 billion), and Instagram (one billion), both also owned by Facebook (Statista.com, 2020). Because they provide different features to their users (Gazit et al., 2019), different Facebook-owned platforms tend to reach individuals with different demographic backgrounds. Regarding gender, Facebook and Instagram show a higher prevalence of female users compared to male users (Perrin and Anderson, 2019), while the WhatsApp audience appears to be more gender-balanced (Statista.com, 2019). Comparing the users between the platforms, Instagram is characterized by a higher prevalence of adolescent and young adult users compared to both the WhatsApp and Facebook platforms (Perrin and Anderson, 2019; Statista.com, 2019).

Personality is an important factor for many life outcomes (for an overview, see Montag and Elhai, 2019), including links to Internet use and its diverse applications (Hamburger and Ben-Artzi, 2000; Kuss et al., 2014; Montag and Reuter, 2017), and is relatively time stable (e.g., Edmonds et al., 2008). Therefore, recent studies have aimed at determining personality’s role in explaining individual preferences for social media use, typically focusing on Big Five personality traits (e.g., Ljepava et al., 2013; Montag et al., 2015b; Brailovskaia and Margraf, 2016; Taber and Whittaker, 2018; Sindermann et al., 2020a). In particular, findings indicate that, compared with non-users, Facebook users report higher scores on extraversion (Ryan and Xenos, 2011; Brailovskaia and Margraf, 2016; Sindermann et al., 2020a), higher neuroticism and openness (Taber and Whittaker, 2018), and lower conscientiousness scores (Ryan and Xenos, 2011; Sindermann et al., 2020a), although the findings are not always consistent across studies. Regarding the association between the Big Five traits and preferences for Instagram and WhatsApp use, existing findings are scant. Although inconclusive, findings from existing studies appear to be in line with those of Facebook users: Instagram users have been found to show higher neuroticism (Gazit et al., 2019) compared to non-users, while WhatsApp users tend to be more extraverted and less conscientious than non-users (Montag et al., 2015b).

Although they provide important findings, the studies mentioned above have mostly only reported findings concerning the associations between personality and individual preferences for a single, specific platform. As such, they failed to investigate differences between groups characterized by different combinations of social media preferences, including those individuals using all the platforms, or none of them. In view of this limitation, in the present study we aim (to our knowledge for the first time) (1) to examine the relative prevalence of groups characterized by different combinations of use of Facebook-owned platforms (i.e., Facebook, Instagram, and WhatsApp) and (2) to investigate whether user groups differ on certain sociodemographic characteristics and Big Five personality traits.

Can we expect significant differences in the distribution of demography and personality across groups of individuals characterized by different patterns of social media use? This question is highly relevant because more and more research is conducted based on samples recruited from these social-media platforms. Thus, it is important to understand whether online samples can be expected to differ on key individual characteristics depending on the platform used for online recruitment. Beyond that, other reasons exist to study the present research question. Currently it is highly debated how social media platforms impact society via filter bubbles and fake news. For instance, Facebook precisely studies the online behavior of each of its users. They do this to be able to present users a personalized news feed, likely to prolong online time on their platform (Montag et al., 2019). This in turn leads to more of a person’s data being monetized by selling the digital profiles of users to the marketing industry (see also Matz et al., 2017). In the realm of politics, liking content of political figures or certain parties could result in radicalization, because users are not confronted with differing world views (Pariser, 2011). Beyond that, fake news is known to be spread via social media (Lazer et al., 2018). Logically, people who abstain from using social media will be less prone to fall for such false information, because they are less likely to get in touch with such news. Although the present work is not able to ultimately answer who falls for fake news (Pennycook and Rand, 2020) or who in particular is prone to the effects of the filter bubble [or echo chamber; see recent work by Sindermann et al. (2020b)], it can at least inform on who decides to use what social media platform or abstains from using them at all.

Although other popular platforms exist beyond those owned by Facebook (e.g., Snapchat, Twitter, China’s WeChat, and Tik-Tok), we decided to focus the present study on Facebook-owned platforms because of their overwhelming reach in terms of number of active users worldwide, whether taken individually or combined together.

## Materials and Methods

### Procedure and Participants

Study data was collected by administering an anonymous questionnaire via an online web survey research platform (SurveyCoder by Christopher Kannen)^1^, and employing a cross-sectional data collection design. As we aimed to recruit a large, demographically heterogeneous sample, the research was advertised by both national and local German-speaking media outlets (TV, radio, press, and Internet). Recruitment was performed using a convenience sampling approach. Participation in the research was voluntary. The survey included questions on demographic variables, use of Facebook-owned platforms, and personality. Participants were informed that the survey would take 20 to 45 min to complete. No monetary reward was offered to participants; however, as an incentive to participate in the present research project, participants were provided with automated, personalized feedbacks on their personality, smartphone, and social media use. All participants were required to provide informed electronic consent prior to participation. Additionally, participants below the age of 18 stated that their legal gardians approved their participation. The study was approved by the local Ethics Committee of Ulm University, Ulm, Germany.

A total of *N* = 3092 German-speaking participants filled in questionnaires on the online platform. All participants reported owning a smartphone. *N* = 89 observations were removed because of missing data on either demographic variables (*N* = 60) or social media use variables (*N* = 29). Eventually, a sample of *N* = 3003 participants (*n* = 1181 males, *n* = 1822 females) remained. The mean age of the sample was 35.53 years (SD = 13.53) with a range from 12 to 79 years. We also collected information about education level (1, no graduation; 2, mainstreamed secondary school; 3, secondary school leaving (graduation) certificate; 4, vocational baccalaureate diploma; 5, A-level/High-school diploma; 6, university of applied sciences degree; 7, university degree). Please refer to the Supplementary Material for details on the distribution of educational level in the present sample (Supplementary Table 3).

### Instruments

#### Use of Facebook-Owned Social Media Platforms

We asked participants to report about use of Facebook-owned social media platforms, i.e., Facebook, Instagram, and WhatsApp. More specifically, we asked participants to indicate if they currently used each platform (yes/no). A large majority (*N* = 2829, 94.21%) reported using at least one of the Facebook-owned platforms, while *N* = 174 (5.79%) reported using none. With *N* = 2762 users (91.97%), WhatsApp was the most widely used platform in the sample, followed by Facebook (*N* = 1733, 57.71%) and Instagram (*N* = 1389, 46.25%; these percentages do not add to 100%, because of non-mutually exclusive item endorsement).

**FIGURE 1:**
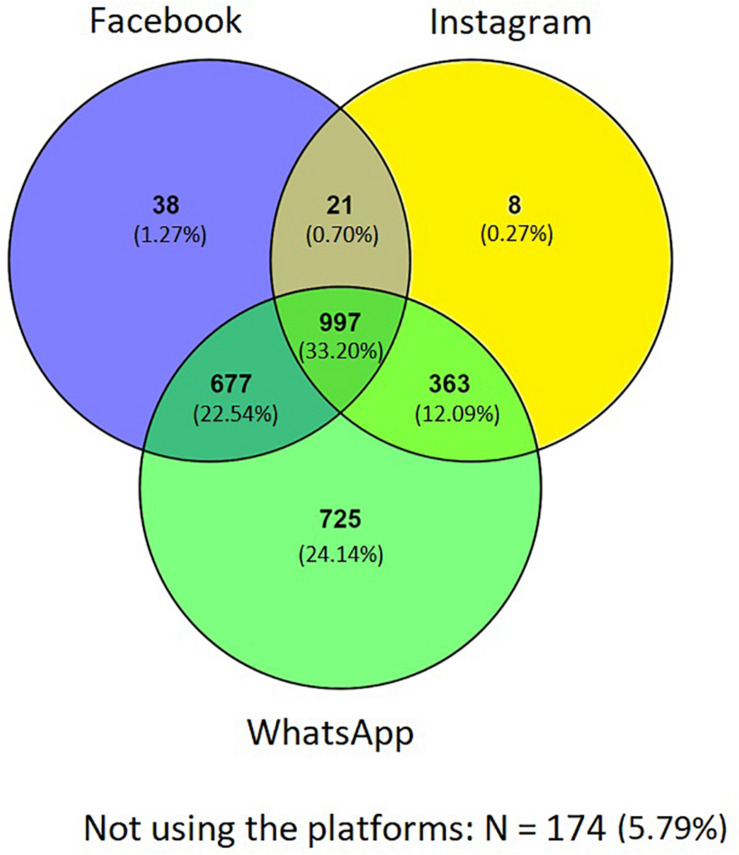
Venn diagram for use of Facebook, Instagram, and WhatsApp in the sample (*N* = 3003).

Figure 1 shows a Venn diagram representing the prevalence of different patterns of social media use in the sample. Among participants reporting use of at least one of the platforms, the majority reported using WhatsApp, either alone (*N* = 725, 24.14% of participants) or in combination with both Facebook and Instagram (*N* = 997, 33.20%), Facebook (*N* = 677, 22.54% of social media users), or Instagram (*N* = 363, 12.09% of participants). Remaining groups were smaller: 38 participants (1.27%) only used Facebook, eight (0.27%) reported only using Instagram, while 21 (0.70%) reported using both Facebook and Instagram.

#### Big Five Inventory

In order to assess individual differences on the Big Five personality traits, we administered the German version of the Big Five Inventory (BFI, Rammstedt and Danner, 2016). The German BFI includes 45 items, including scales to assess Extraversion (8 items), Agreeableness (10 items), Conscientiousness (9 items), Neuroticism (8 items), and Openness (10 items). All items in the questionnaire are answered on a 5-point Likert-Scale ranging from 1 = “very inapplicable” to 5 = “very applicable”. Internal consistency estimates (using Cronbach’s alpha) in the final sample of *N* = 3003 participants were 0.86, 0.74, 0.83, 0.85, and 0.79 for Extraversion, Agreeableness, Conscientiousness, Neuroticism, and Openness, respectively.

In addition to the five broad factors, BFI items can be combined to generate scores for ten facet subscales, two for each trait: Assertiveness (α = 0.82) and Activity (α = 0.57) within the larger Extraversion factor; Altruism (α = 0.54) and Compliance (α = 0.46) within Agreeableness; Order (α = 0.65) and Self-Discipline (α = 0.71) within Conscientiousness; Anxiety (α = 0.76) and Depression (α = 0.57) within Neuroticism; and Aesthetics (α = 0.77) and Ideas (α = 0.57) within Openness. Due to the limited number of items for each subscale, score reliability is known to be modest (Soto and John, 2009; Rammstedt and Danner, 2016).

### Data Analysis

First, we computed descriptive statistics on study variables. We computed means, standard deviations, minimums and maximums for all continuous variables (age, Big Five broad personality traits, and facets), and frequency counts for categorical variables (gender, education level). We present this information in the Supplementary Material (Supplementary Tables 1–4).

Next, we explored associations between different patterns of use of Facebook-owned social media platforms, and both demographic variables (i.e., gender, age, and education level) and personality traits. In order to pursue this aim, we created a multinomial variable grouping individuals by different patterns of social media use. Given the small sample size of participants reporting only using Facebook (*N* = 38), Instagram (*N* = 8), or both Facebook and Instagram (*N* = 21), we decided not to include these groups in the analyses examining between-group differences in demographic variables and personality traits. However, we still report information about the distribution of study variables across all groups in the Supplementary Material (Supplementary Tables 1–3, 5).

Then, one-way analysis of variance (ANOVA) with *post hoc* pairwise comparisons (Bonferroni-corrected) was used to inspect age-related differences by patterns of social media use. A Chi-Square test was used to examine differences in the distribution of demographic variables (gender, educational level) across the groups, while *Z*-tests with a Bonferroni correction were used to perform pairwise multiple comparisons.

Next, we used multivariate analysis of variance (MANOVA) to assess differences in Big Five traits, and their facets, according to different patterns of social media use. First, we performed the analyses by including all five broad personality traits (Extraversion, Agreeableness, Conscientiousness, Neuroticism, and Openness) as dependent variables in a single MANOVA model. Then we performed the same analysis including the ten facets as dependent variables in a single MANOVA model. In both cases, between-group analyses were performed twice. First, we computed the analysis by examining only the effect of the grouping variable distinguishing between different patterns of social media use; next, we computed the analysis by also controlling for the effect of age (continuous covariate), and gender and education level (categorical factors; please note that the interaction between categorical factors were not included in the model). At each step, Wilks’ criterion was used to assess the overall significance of effects. Additionally, to assess pairwise differences between groups within each personality trait, estimated marginal means for each group (both unadjusted and adjusted for other factors and covariates) were compared with Bonferroni-corrected nominal *p-*values (*p* < 0.05). SPSS statistics version 23 was used for all analyses.

## Results

### Association Between Patterns of Social Media Use and Demographic Variables

**FIGURE 2:**
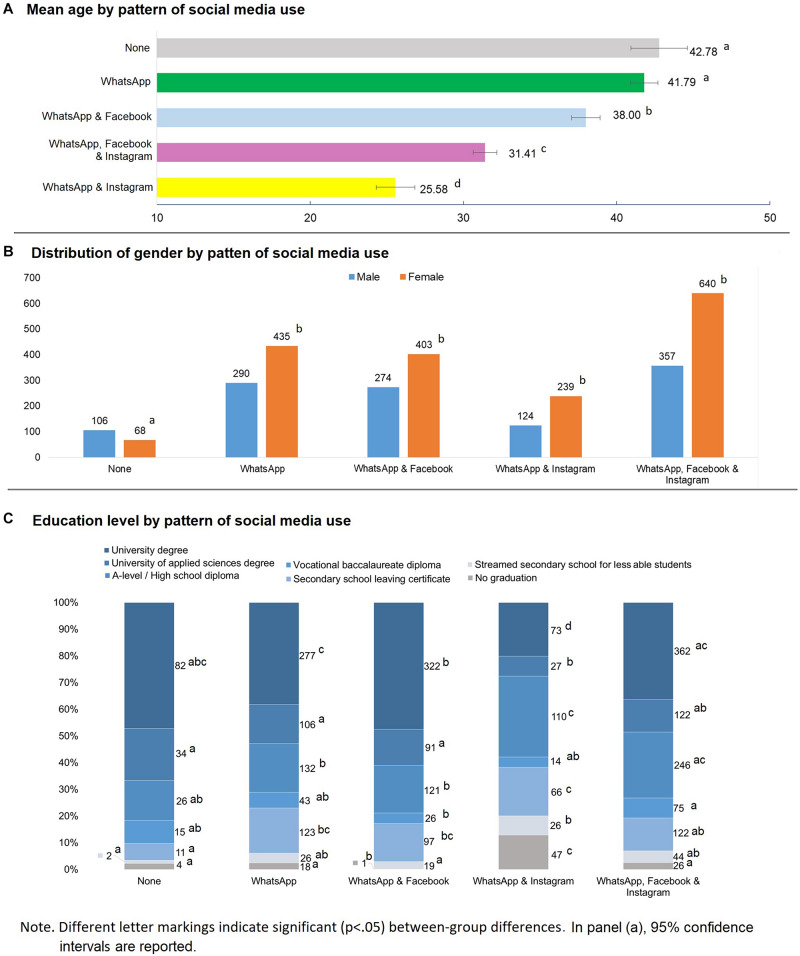
Associations between patterns of social media use and age (Panel **A**), gender **(B)**, and education level **(C)**. In panel **(A)**, 95% confidence interval are reported. Different letter markings indicate significant (*p* < 0.05) differences by pattern of social media use. An example: In Panel **(C)**, prevalence of participants with a university degree is found to differ between the WhatsApp & Facebook group (b marking) and the WhatsApp group (c marking), while both these groups do not differ from the group reporting not using the platforms (abc marking).

Figure 2 provides a visualization of the association between different patterns of social media use and the variables of age, gender, and education levels. With regard to age, one-way ANOVA results supported the existence of strong age-related differences between groups [*F*(4, 2931) = 155.067, *p* < 0.001, η^2^ = 0.175]. *Post hoc* tests (see Figure 2A) indicated that no significant mean age difference existed between participants reporting no social media use and those using only the WhatsApp platform, but these two groups showed higher mean age estimates compared to the remaining groups, which in turn all differed significantly from each other. Among these groups, participants reporting use of Instagram and WhatsApp, but no Facebook use, showed the lowest mean age; those reporting use of both WhatsApp and Facebook showed the highest mean age; and those using all platforms fell in between these two groups of age.

There was a significant association between gender and patterns of social media use (χ^2^ (4) = 43.78, *p* < 0.001, Cramer’s *v* = 0.122). Pairwise comparisons (Figure 2B) indicated that among participants using none of the platforms there was a higher frequency of males than females, while the opposite was found in all other groups. No other significant contrast emerged between the groups.

Finally, we also found a significant association between education level and different patterns of social media use (χ^2^ (24) = 272.156, *p* < 0.001, Cramer’s *v* = 0.152). Pairwise comparisons of education level across patterns of social media use are shown in Figure 2C. We found a higher proportion of individuals holding a university degree among participants reporting using only WhatsApp, or a combination of Facebook and WhatsApp, when compared with participants reporting use of WhatsApp and Instagram. Further, holding a university degree was more prevalent among those using WhatsApp and Facebook when compared to those reporting use of WhatsApp only, and those using all platforms. Interestingly, holding a university degree appeared to be more prevalent among participants using all platforms when compared with those using a combination of only WhatsApp and Instagram (but this is likely confounded with age, because older persons are more likely to hold a higher education degree). Regarding individuals holding a university of applied sciences degree, we found a lower prevalence among those using a combination of WhatsApp and Instagram when compared with all the other groups, except for participants using all platforms. Individuals holding an A-level/high school diploma were more likely to use a combination of WhatsApp and Instagram when compared with all the other groups, except for participants reporting use of all platforms.

Participants reporting use of only WhatsApp, and those using both Facebook and WhatsApp, also showed a lower prevalence of holding an A-level/high school diploma when compared with participants reporting use of all platforms. Individuals holding a vocational baccalaureate diploma were more prevalent among those reporting use of all platforms when compared with those reporting use of WhatsApp and Facebook. Holding a secondary school leaving graduation certificate was more prevalent among participants using a combination of WhatsApp and Instagram than among those using all platforms, and those using none. This latter group also showed a lower prevalence of holding a secondary school leaving certificate than those using only WhatsApp, and those using a combination of WhatsApp and Facebook. Attending mainstreamed secondary school for lesser able students was more prevalent among participants using a combination of WhatsApp and Instagram than those using both WhatsApp and Facebook, and those using none of the platforms. Finally, individuals yet to have graduated were more prevalent among those using both WhatsApp and Instagram than among all the other groups, and showed a lower prevalence among those reporting using WhatsApp and Facebook when compared with all the other groups.

### Differences in Big Five Personality Traits by Pattern of Social Media Use

Next, we explored between-group differences in Big Five personality scores by patterns of social media use. Here, we present results for broad personality traits. Because of the low score reliability found for many of the Big Five personality facets, results concerning facet scores are presented in the Supplementary Material (Supplementaty Presentation 1).

In the MANOVA without control variables, platform use group membership was mildly related to Big Five broad personality traits [Wilks’ λ = 0.953, *F*(20, 9708.71) = 7.032, *p* < 0.001, η^2^ = 0.012]. Significant differences emerged in Extraversion [*F*(4, 2931) = 8.144, *p* < 0.001, η^2^ = 0.011), Conscientiousness [*F*(4, 2931) = 14.949, *p* < 0.001, η^2^ = 0.020], and Neuroticism [*F*(4, 2931) = 5.042, *p* < 0.001, η^2^ = 0.007]. In turn, there were no significant differences in Agreeableness [*F*(4, 2931) = 1.764, *p* = 0.133, η^2^ = 0.002] and Openness [*F*(4, 2931) = 0.972, *p* = 0.422, η^2^ = 0.001] between the groups.

**FIGURE 3:**
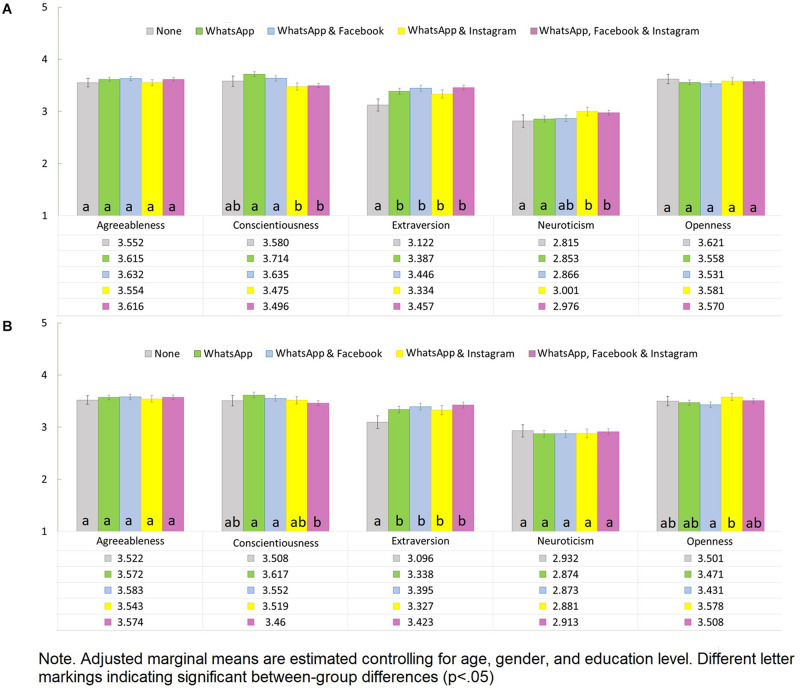
Unadjusted **(A)** and adjusted **(B)** estimated marginal means and 95% confidence intervals for Big Five personality traits by pattern of social media use. Adjusted marginal means are estimated controlling for age, gender, and educational level. Different letter markings indicate significant between-group differences (*p* < 0.05).

Pairwise contrasts comparing estimated marginal means among groups with different patterns of platform use are shown in Figure 3A. Regarding Extraversion, participants reporting not using any of the platforms showed significantly lower scores than all other remaining groups using at least one of the investigated social media platforms. There were no differences in Extraversion across the remaining groups. Regarding Conscientiousness, participants using both WhatsApp and Instagram, as well as those using all the platforms (WhatsApp, Facebook, and Instagram) showed lower scores than those reporting only using WhatsApp, as well as both WhatsApp and Facebook. There were no differences on the Conscientiousness trait between participants reporting no platform use and all the other groups. With respect to Neuroticism, participants using both WhatsApp and Instagram, as well as those using all platforms (WhatsApp, Facebook, and Instagram) had higher scores than those reporting only use of WhatsApp, or no platform at all. There were no differences between participants reporting both WhatsApp and Facebook, compared to the other groups.

When including control variables in the MANOVA, the overall relationship between different patterns of social media use and personality traits was reduced [Wilks’ λ = 0.972, *F*(20, 9682.18) = 4.200, *p* < 0.001, η^2^ = 0.007]. Among the control variables, gender showed a large effect [Wilks’ λ = 0.849, *F*(5, 2919) = 103.721, *p* < 0.001,η^2^ = 0.151]. Education level [Wilks’ λ = 0.952, *F*(30, 11678.00) = 4.802, *p* < 0.001, η^2^ = 0.010] and age [Wilks’ λ = 0.980, *F*(5, 2919.00) = 12.215, *p* < 0.001, η^2^ = 0.020] also showed significant effects. In the adjusted model, differences in social media use were still related to differences in Extraversion [*F*(4, 2923) = 6.995, *p* < 0.001, η^2^ = 0.009], and Conscientiousness [*F*(4, 2923) = 5.925, *p* < 0.001, η^2^ = 0.008]. However, after including control variables, there were no between-group differences on Agreeableness [*F*(4, 2923) = 0.658, *p* = 0.621, η^2^ < 0.001] and Neuroticism [*F*(4, 2923) = 0.508, *p* = 0.730, η^2^ < 0.001], while a new, small between-group effect emerged for the Openness trait [*F*(4, 2923) = 3.508, *p* = 0.007, η^2^ = 0.005]. Estimated marginal means were then subjected to pairwise comparisons. Regarding Extraversion, the overall pattern was the same found when inspecting the unadjusted contrasts (Figure 3B). Regarding Conscientiousness, results were similar to those observed in the unadjusted contrasts, except for the group of participants using both WhatsApp and Instagram, which no longer showed a significant mean difference between the groups using only WhatsApp, or both WhatsApp and Facebook. Regarding Openness, the group of participants using both WhatsApp and Instagram reported higher scores than participants only using WhatsApp and Facebook, while other contrasts were not significant.

## Discussion

The present work aimed at (1) investigating the prevalence of groups of social-media users characterized by different combinations of use of Facebook-owned social media platforms, namely Facebook, WhatsApp, and Instagram, and (2) examining differences in sociodemographic characteristics and personality traits among emerging groups. To pursue this aim, we grouped individuals based on emerging combinations of self-reported social media preferences, and evaluated the significance of between-group differences in the distributions of gender, age, education levels, and Big-Five personality traits. We believe this study to be of relevance, because most previous studies exclusively focused on one social media platform, yet many users spend their time on different social media platforms. Hence, the nature of social media use is more complex than many studies suggest.

Concerning specific platforms use prevalence, we found that 91.97% of the sample used WhatsApp, making it the most used platform (a finding that fits previous findings, Montag et al., 2015b), followed by Facebook (57.71%), and Instagram (46.25%). Instagram use was in general scarcer, reflecting also international user numbers (Statista.com, 2020). Regarding the prevalence of different combinations of social media use, our data show that the largest group of individuals was the one using all Facebook owned platforms (33.20%). Other frequent combinations of social media use resulted in a group of people only using WhatsApp (24.14%), a combination of WhatsApp and Facebook (22.54%), and a combination of WhatsApp and Instagram (12.09%). Overall, the vast majority of Facebook and Instagram users also used at least one of the other Facebook-owned social media platforms (99.42% of Instagram users; 97.81% of Facebook users), while WhatsApp users were less likely to use other Facebook-owned platforms (73.75% of WhatsApp users).

Next, we found that sociodemographic variables such as age and gender varied significantly according to the specific pattern of social media use reported by participants. In our study, age showed the strongest association with individuals’ social media use, with non-users and WhatsApp users being the oldest group, and participants using both WhatsApp and Instagram being the youngest. Overall, our findings support the assumption that Instagram attracts the younger user generation. Further, our findings show that females are more strongly represented on social media – no matter which social media combination is investigated, which is also coherent with previous findings (e.g., Andreassen, 2015). The education findings are not further discussed now, because we believe them to be strongly confounded with age. To illustrate this point, Instagram use is more often reported in groups with lower education levels, but these persons are also younger – hence are still often attending school.

From a personality psychology perspective, findings from the present study clearly highlight that social media users differ in extraversion from non-users. This is the most robust finding with respect to the Big Five, also underlining the idea that extraverts have a stronger need for social interaction, which might result in them choosing to use social media to communicate with others via this digital channel in order to fulfill their needs for bonding. Both Extraversion facets drive this effect (see Supplementary Material, Supplementary Presentation 1), with non-users reporting to both be less active and assertive when compared with social media users.

We also found that individuals using all social media platforms or simply WhatsApp and Instagram (hence also the younger persons; see Figure 2A) had the lowest conscientiousness scores. This is not surprising, as low conscientiousness is known to have a direct relationship with tendencies toward Internet Use Disorders, which in turn are strongly characterized by social media use (Montag et al., 2015a; Müller et al., 2017; Sha et al., 2019). Supplementary analyses showed this effect was especially pronounced for Conscientiousness’ order facet; hence, individuals using all Facebook-owned platforms tend to be less orderly and diligent.

Finally, we found Neuroticism was significantly higher among individuals using all platforms, or just WhatsApp and Instagram, compared to those reporting using no platform, or just WhatsApp. Interestingly, the association between Neuroticism and social media use when controlling for demographic variables would suggest an underlying confounding effect.

Overall, findings from the present study highlight the role of Extraversion in explaining differences in social media use vs. non use. These findings are coherent with those reported by studies examining Big Five personality traits and use of specific social media platforms (e.g., Ryan and Xenos, 2011; Montag et al., 2015b; Brailovskaia and Margraf, 2016; Sindermann et al., 2020a). Further, going beyond previous studies, we found that the Neuroticism and Conscientiousness traits were also related to the combined use of different social media platforms, which in turn suggests that distinct links emerging from previous studies may well reflect a common underlying association between these traits and individual differences in the inclination toward social media use. Still, it is worthy to note that although the described personality associations fit well with the literature, between-group differences were generally small-sized and could be detected due to the large sample size.

### Limitations

The present study has several limitations, which need to be addressed. First, our sampling strategy used to recruit participants involved convenience sampling. Hence, limited inference can be derived from the present study regarding the actual prevalence of different patterns of social media use in the reference population. However, because of the large sample recruited, results about the association between emerging different patterns of social media use and both demographic variables, and personality traits, appear to be quite robust. An additional limit of the present study relates to its focus only on Facebook-owned platforms. Nevertheless, we believe the focus to be relevant, because these platforms are currently the most successful and important ones in terms of numbers of active users (Statista.com, 2019). Still, other platforms such as Snapchat, Twitter, or recently also Chinese platforms including WeChat (Montag et al., 2018) and TikTok, have been steadily increasing in popularity. The correlational nature of the present work represents another relevant limitation, preventing us from obtaining insight into cause-effect mechanisms or how personality relates to different types of platform usage (hence activity patterns). Finally, associations between facet levels of the Big Five and social media usage need to be handled with caution, as internal consistency was in the lower range of acceptability. Despite these shortcomings the present work is much needed, because it provides insight from a bird’s eye view on who has an account on the different social media platforms owned by Facebook.

### Conclusion

In sum, the present work shows social media users of Facebook’s platforms to be younger, more likely female, and more extraverted compared to non-users. Our findings might be of relevance when research is carried out via social media platforms, because sample characteristics might be biased. Extending previous findings (e.g., Rife et al., 2016), we found that significant differences in the observed distributions of demographics and personality traits can be observed depending on the specific combination of social media platforms used by participants. These findings have practical implications, because biases in the distributions of demographics and personality may affect results concerning the distribution of variables of interest, as well as their associations (e.g., behaviors, Dash et al., 2019; intelligence, Jacobs et al., 2012); abilities, Soh and Jacobs, 2013; and health, Feldman et al., 1999). Our findings might also be of relevance when aiming to understand who is at risk of falling for fake news. Logically, participants who abstain from using social media apps should receive less contact with fake news that are shared online. With respect to filter bubbles, it is highly interesting that Sindermann et al. (2020b) recently observed that low conscientious and high neurotic people have higher tendencies to also inform themselves only via the news feed on social media. Fittingly, those persons are also those who more likely end up using multiple social media applications.

## Data Availability Statement

The original contributions presented in the study are included in the Supplementary Material.

## Ethics Statement

The studies involving human participants were reviewed and approved by the Ethic Committee of Ulm University.

## Author Contributions

CM designed the present study. DM analyzed the data and wrote the “Materials and Methods” and “Results” sections. CM drafted the “Introduction” and “Discussion” sections, which were later edited and revised by DM, JE, and CS. CS checked independently all statistics. All authors worked over the manuscript and critically revised it.

## Conflict of Interest

CM mentions that he is part of a discussion circle (Digitalität und Verantwortung: https://about.fb.com/de/news/h/gespraechskreis-digitalitaet-und-verantwortung/) debating ethical questions linked to social media, digitalization and society/democracy at Facebook. In this context, he receives no salary for his activities. Finally, CM currently is an independent researcher on the scientific advisory board of the Nymphenburg group. For this activity he is financially compensated. The remaining authors declare that the research was conducted in the absence of any commercial or financial relationships that could be construed as a potential conflict of interest.

## Footnotes

1 https://ckannen.com/
