# Supplementary material for: One Social Media Company to Rule Them All: Associations Between Use of Facebook-Owned Social Media Platforms, Sociodemographic Characteristics, and the Big Five Personality Traits
Source: Front Psychol. 2020 May 29;11:936. doi: 10.3389/fpsyg.2020.00936 (PMC7273309; doi:10.3389/fpsyg.2020.00936)
Supplement: Supplementary file 1 [file Table_1.docx]

Supplementary Material

**Table 1. Descriptive statistics for age in the whole sample, and by pattern of social media use**

| Variable | N | Minimum | Maximum | Mean | Std. Deviation |
| --- | --- | --- | --- | --- | --- |
| Whole sample | 3003 | 12.00 | 79.00 | 35.53 | 13.53 |
| Pattern of Social Media Use |  |  |  |  |  |
| None | 174 | 12.00 | 71.00 | 42.78 | 11.86 |
| WhatsApp | 725 | 12.00 | 79.00 | 41.79 | 13.95 |
| WhatsApp & Facebook | 677 | 12.00 | 79.00 | 38.00 | 11.37 |
| WhatsApp & Instagram | 363 | 12.00 | 69.00 | 25.58 | 13.06 |
| WhatsApp, Facebook & Instagram | 997 | 13.00 | 76.00 | 31.41 | 11.53 |
| Facebook & Instagram | 21 | 23.00 | 48.00 | 36.00 | 7.91 |
| Facebook | 38 | 22.00 | 72.00 | 41.18 | 11.08 |
| Instagram | 8 | 14.00 | 54.00 | 38.00 | 14.86 |
