# Supplementary material for: One Social Media Company to Rule Them All: Associations Between Use of Facebook-Owned Social Media Platforms, Sociodemographic Characteristics, and the Big Five Personality Traits
Source: Front Psychol. 2020 May 29;11:936. doi: 10.3389/fpsyg.2020.00936 (PMC7273309; doi:10.3389/fpsyg.2020.00936)
Supplement: Supplementary file 2 [file Table_2.docx]

Supplementary Material

**Table 2. Distribution of gender in the whole sample, and by pattern of social media use**

|  | Pattern of Social Media Use | | | | | | | | |
| --- | --- | --- | --- | --- | --- | --- | --- | --- | --- |
|  | None | WhatsApp | WhatsApp & Facebook | WhatsApp & Instagram | WhatsApp,  Facebook & Instagram | Facebook & Instagram | Facebook | Instagram | Total |
| Male | 106 | 290 | 274 | 124 | 357 | 8 | 17 | 5 | 1181 |
| Female | 68 | 435 | 403 | 239 | 640 | 13 | 21 | 3 | 1822 |
