# Supplementary material for: One Social Media Company to Rule Them All: Associations Between Use of Facebook-Owned Social Media Platforms, Sociodemographic Characteristics, and the Big Five Personality Traits
Source: Front Psychol. 2020 May 29;11:936. doi: 10.3389/fpsyg.2020.00936 (PMC7273309; doi:10.3389/fpsyg.2020.00936)
Supplement: Supplementary file 3 [file Table_3.docx]

Supplementary Material

**Table 3. Distribution of education level in the whole sample, and by pattern of social media use**

|  | Pattern of Social Media Use | | | | | | | | | |
| --- | --- | --- | --- | --- | --- | --- | --- | --- | --- | --- |
| Education level | None | WhatsApp | WhatsApp & Facebook | WhatsApp & Instagram | WhatsApp,  Facebook & Instagram | Facebook & Instagram | Facebook | Instagram | Total | |
| 1 = no graduation | 4 | 18 | 1 | 47 | 26 | 0 | 0 | 0 | 96 | |
| 2 = mainstreamed secondary school for lesser able students | 2 | 26 | 19 | 26 | 44 | 0 | 0 | 0 | 117 | |
| 3 = secondary school leaving certificate | 11 | 123 | 97 | 66 | 122 | 1 | 3 | 0 | 423 | |
| 4 = vocational baccalaureate diploma | 15 | 43 | 26 | 14 | 75 | 0 | 4 | 0 | 177 | |
| 5 = A-level / High school diploma | 26 | 132 | 121 | 110 | 246 | 3 | 5 | 3 | 646 | |
| 6 = university of applied sciences degree | 34 | 106 | 91 | 27 | 122 | 3 | 1 | 0 | 384 | |
| 7 = university degree | 82 | 277 | 322 | 73 | 362 | 14 | 25 | 5 | 1160 |  |
