# Supplementary material for: One Social Media Company to Rule Them All: Associations Between Use of Facebook-Owned Social Media Platforms, Sociodemographic Characteristics, and the Big Five Personality Traits
Source: Front Psychol. 2020 May 29;11:936. doi: 10.3389/fpsyg.2020.00936 (PMC7273309; doi:10.3389/fpsyg.2020.00936)
Supplement: Supplementary file 4 [file Table_4.docx]

Supplementary Material

**Table 4. Descriptive statistics for the BFI: personality traits and facets (N = 3003)**

| Variable | Minimum | Maximum | Mean | Std. Deviation |
| --- | --- | --- | --- | --- |
| Extraversion | 1.00 | 5.00 | 3.40 | 0.78 |
| Assertiveness | 1.00 | 5.00 | 3.38 | 0.88 |
| Activity | 1.00 | 5.00 | 3.48 | 0.82 |
| Agreeableness | 1.30 | 5.00 | 3.61 | 0.55 |
| Altruism | 1.00 | 5.00 | 3.59 | 0.63 |
| Compliance | 1.00 | 5.00 | 3.48 | 0.72 |
| Conscientiousness | 1.00 | 5.00 | 3.58 | 0.67 |
| Order | 1.00 | 5.00 | 3.36 | 1.04 |
| Self-Discipline | 1.00 | 5.00 | 3.54 | 0.69 |
| Neuroticism | 1.00 | 5.00 | 2.91 | 0.79 |
| Anxiety | 1.00 | 5.00 | 2.99 | 0.87 |
| Depression | 1.00 | 5.00 | 2.70 | 0.97 |
| Openness | 1.30 | 5.00 | 3.57 | 0.62 |
| Aesthetics | 1.00 | 5.00 | 3.48 | 0.97 |
| Ideas | 1.40 | 5.00 | 3.59 | 0.60 |
