# Supplementary material for: One Social Media Company to Rule Them All: Associations Between Use of Facebook-Owned Social Media Platforms, Sociodemographic Characteristics, and the Big Five Personality Traits
Source: Front Psychol. 2020 May 29;11:936. doi: 10.3389/fpsyg.2020.00936 (PMC7273309; doi:10.3389/fpsyg.2020.00936)
Supplement: Supplementary file 5 [file Table_5.docx]

Supplementary Material

**Table 5. Mean and standard deviation for BFI personality traits and their facets by pattern of social media use (N = 3003)**

|  | Pattern of Social Media Use | | | | | | | | | | | | | | | |  |
| --- | --- | --- | --- | --- | --- | --- | --- | --- | --- | --- | --- | --- | --- | --- | --- | --- | --- |
|  | None | | WhatsApp | | WhatsApp  & Facebook | | WhatsApp  & Instagram | | WhatsApp,  Facebook & Instagram | | Facebook  & Instagram | | Facebook | | Instagram | |  |
|  | M | SD | M | SD | M | SD | M | SD | M | SD | M | SD | M | SD | M | SD |  |
| Extraversion | 3.12 | 0.82 | 3.39 | 0.77 | 3.45 | 0.77 | 3.33 | 0.78 | 3.46 | 0.79 | 3.77 | 0.71 | 3.34 | 0.78 | 3.53 | 0.78 |  |
| Assertiveness | 3.08 | 0.88 | 3.36 | 0.87 | 3.42 | 0.86 | 3.31 | 0.89 | 3.42 | 0.89 | 3.76 | 0.82 | 3.32 | 0.87 | 3.45 | 0.86 |  |
| Activity | 3.31 | 0.85 | 3.47 | 0.80 | 3.50 | 0.80 | 3.41 | 0.84 | 3.52 | 0.83 | 3.83 | 0.75 | 3.43 | 0.78 | 3.81 | 1.00 |  |
| Agreeableness | 3.55 | 0.50 | 3.62 | 0.54 | 3.63 | 0.53 | 3.55 | 0.59 | 3.62 | 0.55 | 3.61 | 0.61 | 3.51 | 0.64 | 3.08 | 0.88 |  |
| Altruism | 3.49 | 0.58 | 3.56 | 0.63 | 3.61 | 0.61 | 3.60 | 0.62 | 3.62 | 0.63 | 3.76 | 0.45 | 3.38 | 0.73 | 3.19 | 0.93 |  |
| Compliance | 3.47 | 0.69 | 3.51 | 0.72 | 3.48 | 0.69 | 3.46 | 0.78 | 3.48 | 0.72 | 3.27 | 0.89 | 3.31 | 0.72 | 3.04 | 1.12 |  |
| Conscientiousness | 3.58 | 0.67 | 3.71 | 0.65 | 3.64 | 0.63 | 3.48 | 0.71 | 3.50 | 0.68 | 3.63 | 0.57 | 3.57 | 0.79 | 3.54 | 0.62 |  |
| Order | 3.36 | 1.05 | 3.50 | 1.01 | 3.41 | 1.02 | 3.28 | 1.08 | 3.26 | 1.04 | 3.33 | 0.98 | 3.26 | 1.25 | 3.13 | 1.16 |  |
| Self-Discipline | 3.54 | 0.68 | 3.68 | 0.66 | 3.59 | 0.65 | 3.43 | 0.72 | 3.43 | 0.70 | 3.65 | 0.63 | 3.53 | 0.76 | 3.55 | 0.45 |  |
| Neuroticism | 2.82 | 0.81 | 2.85 | 0.77 | 2.87 | 0.80 | 3.00 | 0.77 | 2.98 | 0.80 | 2.63 | 0.93 | 2.69 | 0.76 | 2.83 | 0.69 |  |
| Anxiety | 2.87 | 0.85 | 2.94 | 0.84 | 2.96 | 0.87 | 3.09 | 0.87 | 3.06 | 0.88 | 2.73 | 1.05 | 2.73 | 0.92 | 2.53 | 0.74 |  |
| Depression | 2.63 | 1.03 | 2.60 | 0.95 | 2.63 | 0.94 | 2.84 | 0.97 | 2.81 | 0.97 | 2.24 | 1.20 | 2.55 | 0.95 | 3.06 | 0.98 |  |
| Openness | 3.62 | 0.56 | 3.56 | 0.60 | 3.53 | 0.62 | 3.58 | 0.62 | 3.57 | 0.63 | 3.85 | 0.80 | 3.75 | 0.65 | 3.71 | 0.40 |  |
| Aesthetics | 3.41 | 0.89 | 3.45 | 0.96 | 3.41 | 0.96 | 3.56 | 1.00 | 3.52 | 0.98 | 3.90 | 0.94 | 3.53 | 0.95 | 3.67 | 0.59 |  |
| Ideas | 3.72 | 0.61 | 3.60 | 0.59 | 3.58 | 0.61 | 3.55 | 0.58 | 3.58 | 0.59 | 3.75 | 0.83 | 3.84 | 0.61 | 3.73 | 0.32 |  |
