# Supplementary material for: One Social Media Company to Rule Them All: Associations Between Use of Facebook-Owned Social Media Platforms, Sociodemographic Characteristics, and the Big Five Personality Traits
Source: Front Psychol. 2020 May 29;11:936. doi: 10.3389/fpsyg.2020.00936 (PMC7273309; doi:10.3389/fpsyg.2020.00936)
Supplement: Supplementary file 8 [file Presentation_1.pdf]

Supplementary Material

Differences in Big Five Personality Facets by Pattern of Social Media Use

Results of MANOVA showed that group differences in patterns of platform use were related to small differences in personality facets (Wilks' λ=0.937, F(40, 11081.74)=4.790, p<.001, $\eta^{2=}$.016). There were significant differences among all facets, except for the facets of Agreeableness (Altruism: F(4, 2931)=1.995, p=.093, η^2=.003; Compliance: F (4, 2931)=0.354, p=.841, η^2=.000). Results of pairwise comparisons are shown in Figure 4, panel a. Regarding Extraversion facets (Assertiveness: F(4, 2931)=6.576, p <.001, η^2= .009; Activity: F(4, 2931)=3.337, p=.010, η^2=.005), pairwise comparisons showed participants not using the social media had lower Assertiveness scores when compared with the other groups (i.e., participants reporting different combinations of social media use); concerning the Activity facet, participants not using the platforms showed lower scores than participants using all platforms, while no other between-group differences emerged.

Regarding the facets of Conscientiousness (Order: F(4, 2931)=6.812, p<.001, η^2=.009, Self-Discipline: F(4, 2931)=16.935, p <.001, η^2=.023), we see that participants reporting using only WhatsApp show higher scores on Order when compared with participants using either a combination of WhatsApp and Instagram, or using all platforms. Participants reporting using a combination of WhatsApp and Facebook use also showed higher Order scores than those reporting using all platforms. For the Self-Discipline facet, participants using only WhatsApp or a combination of WhatsApp and Facebook use show higher scores than those reporting using either a combination of WhatsApp and Instagram, or those who use all platforms.

Concerning Neuroticism facets (Anxiety: F(4, 2931)=4.348, p=.002, η^2=.006; Depression: F(4, 2931)=8.173, p<.001, η^2=.011), pairwise comparisons did not reveal significant between group differences on the Anxiety facet; in turn, we note that participants reporting using either a combination of WhatsApp and Instagram, or those who use all platforms have higher Depression scores compared with participants using only WhatsApp, or a combination of WhatsApp and Facebook.

Finally, regarding Openness facets (Aesthetics: F(4, 2931)=2.405, p=.048, η^2= .003; Ideas: F(4, 2931)=2.729, p=.028, η^2=.004), pairwise comparisons did not reveal significant between group differences on the Aesthetics facet; in turn, we see that participants reporting not using any of the platforms have higher Ideas scores when compared to participants using all the patterns of social media use, except for those reporting only using WhatsApp.

When including control variables in the MANOVA, the overall relationship between different patterns of social media use and personality facets was again reduced (Wilks' λ=0.960, F(40, 11051.40)=2.961, p<.001, $\eta^{2=}$.010). Among the control variables, gender did show a large effect (Wilks' λ=0.813, F(10, 2914)=67.056, p< .001,$\eta^{2=}$.187). Education level (Wilks' λ=.931, F(60, 15272.41)=3.508, p<.001, $\eta^{2=}$.012) and age (Wilks' λ=.961, F(10, 2914.00)=11.936, p<.001, $\eta^{2=}$.039) also showed significant effects. In the adjusted model differences in social media use were still related to

differences in Extraversion facets (Assertiveness: F(4, 2923)=5.994, p < .001, η^{2}=.008; Activity: F(4, 2923)=2.664, p= .031, η^{2}=.004), Conscientiousness facets (Order: F(4, 2923)=2.872, p=.022, η^{2}=.004, Self-Discipline: F(4, 2923)=7.106, p <.001, η^{2}=.010), and the Aesthetics, but not the Ideas facet of Openness (Aesthetics: F(4, 2923)=5.596, p<.001, η^{2}=.008; Ideas: F(4, 2923)=1.481, p= .205, η^{2} =.002).

In turn, there were no significant differences on the facets of Agreeableness (Altruism: F(4, 2923)=1.322, p=.259, η^{2}=.002; Compliance: F(4, 2923)=0.656, p=.622, η^{2}<.001) and Neuroticism (Anxiety: F(4, 2923)=0.182, =.948, η^{2}<.001; Depression: F(4, 2923)=2.065, p=.083, η^{2}=.003).

Results of pairwise comparisons are shown in Figure 1, panel b. The Extraversion facets showed the same pattern of between-group differences as in the unadjusted model. For Conscientiousness facets, patterns were also similar but we note a few small differences: on the Order facets, participants reporting use of both WhatsApp and Facebook no longer differ from those reporting using all platforms; on the Self-Discipline facet, participants reporting use of both WhatsApp and Facebook no longer differ from those reporting using a combination of WhatsApp and Instagram. Finally, regarding the Aesthetics facet of Openness, we see that participants reporting use of a combination of WhatsApp and Instagram have higher scores than those reporting using only WhatsApp; additionally, participants reporting using all platforms shows also higher Aesthetics scores than those using a combination of WhatsApp and Facebook.

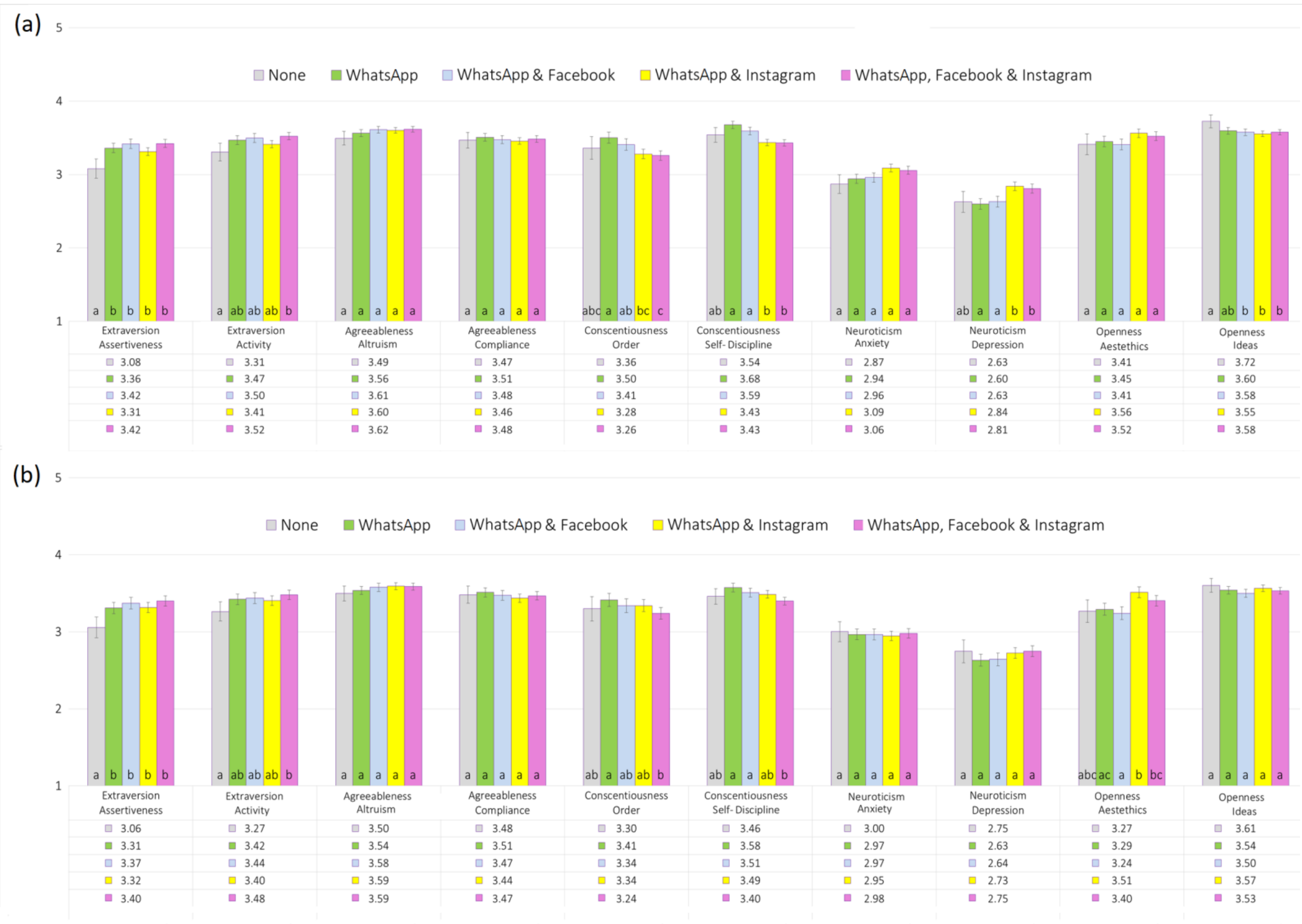

Note. Adjusted marginal means (b) are estimated controlling for age, gender, and education level. Different letter markings indicating significant between-group differences (p<.05)

Figure 1. Unadjusted (a) and adjusted (b) estimated marginal means for Big Five personality facets by pattern of social media use
